# “The More You Give the Wearable, the More It Gives You”: How Regular Exercisers Navigate Exercise Using Wearable Devices

**DOI:** 10.1007/s10484-025-09762-6

**Published:** 2026-01-05

**Authors:** Adam H. Ibrahim, Cory T. Beaumont, Kelley Strohacker

**Affiliations:** 1 Department of Kinesiology, Recreation, and Sport Studies, The University of Tennessee, Knoxville, Knoxville, TN USA; 2 Division of Kinesiology and Health, University of Wyoming, 111 Corbett Building, Laramie, WY USA; 3 Department of Allied Health, Sport, and Wellness, Baldwin Wallace University, Berea, OH USA

**Keywords:** Wearable devices, Behavioral insights, Heart rate variability, Self-monitoring

## Abstract

The increased use of consumer off-the-shelf wearables (COTSW) for exercise training and self-monitoring has resulted in some COTSW offering individual ‘recovery’ or ‘readiness’ scores. These scores are derived using heart rate variability (HRV) and are designed to be easily interpreted (percent score; color codes) to assist users in optimizing their training. Limited research exists on the perceived informational utility associated with HRV-derived scores, which can impact user adoption and fidelity. The purpose of this qualitative study was to understand individuals’ experiences using and interacting with their COTSW and readiness/recovery scores. Seventeen regular exercisers who owned and used a Whoop™ band or Oura™ ring for at least 3 months participated in a one-on-one virtual semi-structured interview. Interviews were analyzed using reflexive thematic analysis, with themes supported by ‘in-vivo’ quotes. This paper focuses on three key themes for a comprehensive demonstration. Theme 1, *‘It’s kinda validating*, *right?’* (Detroit) highlights how users found validation and reinforcement in their lifestyle choices and feelings about their well-being. Theme 2, *‘It’s really putting the power in my hands*,’ (Misty) indicates users’ curiosity and experimentation with their devices, leading to behavioral adjustments aimed at improving their readiness/recovery scores. Theme 3, potential for *‘more of an emotional response rather than a rational one*, (Brian) demonstrates the complex reactive responses users may have towards these scores and each wearable’s interface. These findings contribute to the understanding of how COTSW estimates of readiness or recovery impact users’ behavior and self-perception, while highlighting the potential need for user education and design considerations to promote positive interactions with such data.

## Introduction

The adage “what gets measured gets managed” (Drucker, 1994) underscores the critical role of tracking and documenting engagement engaging in target behaviors and achieving goals, particularly in the realm of exercise and fitness. This concept of self-monitoring, which traditionally refers broadly to keeping a record of a specified behavior (Michie et al., 2011), often includes details about timing, type, duration and/or intensity. Self-monitoring has been recognized as an effective strategy for a variety of behaviors and outcomes, including weight loss (Burke et al., 2011; Vanwormer et al., 2008), eating behaviors (Martinelli et al., 2020), and exercise performance (Bourdon et al., 2017). Its counterpart, journaling involves the structured recording of thoughts, behaviors, and experiences related to a target behavior (e.g., exercise) and can provide deeper insight into the context surrounding behavior patterns (Hubbs & Brand, 2005). However, implementing monitoring requires an investment of time, financial, and human resources to obtain, analyze, and utilize the data effectively (Burke et al., 2009; Orji et al., 2018).

The use of consumer off-the-shelf wearables (COTSW) as tools for self-monitoring training and guiding training has significantly increased (Bunn et al., 2018), with advances in sensor technology enhancing precision in data collection. These improvements, coupled with wearables’ increased portability and user-friendly interfaces, (Chan et al., 2012; Mukhopadhyay, 2015), allow for sophisticated data analyses that provide users with valuable insights into aspects of their personal health including stress levels, as well as physical, cognitive, and emotional states (Aroganam et al., 2019; Bunn et al., 2018; Peake et al., 2018). Heart rate variability (HRV), a key physiological metric, is frequently recorded due to its versatility in reflecting various states (Shaffer & Ginsberg, 2017). Modern wearables now integrate individual HRV-derived scores into algorithms designed to provide metrics that are purported to be easily interpreted, often labeled as ‘recovery’ or ‘readiness’ values to assist users in optimizing their training on a session-by-session basis (Bunn et al., 2018; Lamkin, 2018). Adjusting training in this manner aligns with expert-led calls for person-adaptive approaches for physical activity (Chevance et al., 2021; Conroy et al., 2020; de Souto Barreto, 2013), and for sports-specific training (Kiely, 2012, 2018).

Routinely assessing pre-exercise readiness or recovery states has potentially beneficial implications for how exercise is performed and experienced (Strohacker et al., 2024). Strohacker and colleagues (2024) align exercise-related readiness with the positioning of physical, mental, and emotional resources as contributors to health-related behavior maintenance. Recovery, on the hand, has been viewed as the “post-exercise window of opportunity,” (p.3; Luttrell & Halliwill, 2015) involving the processes that restore the body and mind after exercise, including optimizing sleep, macronutrient intake, and various body integrity-enhancing techniques (Bezuglov et al., 2021; Dupuy et al., 2018). An emerging body of research suggests that subjective readiness contribute to experiential outcomes (i.e., affective responses; Beaumont et al., 2021; Rose & Parfitt, 2010; Smith-Ricketts et al., 2023; Strohacker et al., 2019). Optimizing experiential outcomes (i.e., minimizing the potential for negative responses) is critical as more positive affective responses are considered a driving factor of physical activity and exercise engagement (Brand & Ekkekakis, 2017, 2019).

Despite the growing popularity of COTSWs among individuals engaging in exercise training, there remains a significant gap in scientific research concerning the perceived informational utility and resulting actions associated with the use of HRV-derived readiness and recovery scores. There is a distinct need to address this gap, as perceptions of utility and satisfaction with outcomes influence users’ adoption and continued use of the device, including compliance with suggested actions from the device (e.g., prioritize recovery, your low HRV balance signals that your body may need time to recover today). Optimizing such outcomes would be further useful for researchers intending to integrate wearable devices within behavior change interventions targeting physical activity and exercise (Ibrahim et al., 2024a, b). In a prior publication (Ibrahim et al., 2024b), initial results from the parent study were presented, which demonstrated that individuals do use their COTSW scores to adjust their training, as well as guide other lifestyle behaviors to then enhance their ‘recovery’ or ‘readiness’ scores, while understanding that these scores have important limitations (Ibrahim et al., 2024b). This current paper aims to present the remaining themes from the initial investigation, which details the processes that enable or potentially thwart COTSW engagement and resultant behaviors.

## Methods

### Design Overview

In accordance with the ethical guidelines set forth by the University’s Institutional Review Board, all procedures described in this study received approval prior to participant recruitment and data collection. This study employed a descriptive qualitative approach (Sandelowski, 2000, 2010) via one-on-one semi-structed interviews to gain insight into the perceptions and experiences of individuals utilizing COTSW’s that display HRV-derived scores. This methodological approach is deemed suitable for exploring phenomenon with limited knowledge, enabling researchers to derive meaning from the language and terminology used by individual during the analysis process. Within the framework of a constructivist paradigm (Denzin & Lincoln, 2011; Doyle et al., 2020), we acknowledged the existence of valid diverse realities and recognized that individuals construct their own subjective realities. Due to the abundance of data and the limitations imposed by word constraints, this paper prioritizes the exploration of the remaining three themes the parent study. This approach ensures that each theme received the necessary attention and space for a comprehensive examination, aligning with the objectives of our research endeavor. The primary research procedures and analysis approach are summarized below; additional methodological details can be found in the initial publication (Ibrahim et al., 2024b).

### Procedure

#### Reflexivity of the Researchers

The lead author, a male doctoral student, served as the primary interviewer during this study and has had extensive interactions with individuals regarding the usage of COTSW monitoring readiness and recovery. He has completed graduate-level coursework focused within attaining a qualitative research certificate. Throughout the study, he received support and guidance from the team and other experienced qualitative researchers to enhance the overall quality of the investigation.

#### Participants and Recruitment

Recruitment for interested individuals was conducted through social media (Instagram and Facebook), flier advertisements posted at a Southeastern flagship university and nearby gyms, and snowball sampling via participant referrals. Inclusion criteria were as follows: aged 18–45 years old, access to a computer with a camera and microphone (for virtual interview purposes), ownership of, and self-reported utilization of a Whoop™ band or Oura™ Ring for at least 3 months for a minimum of 4 days per week. The selection of these particular COSTWs was based on their substantial market share, amounting to 6.15 billion dollars and present recovery or readiness scores to users (Gentrup, 2022; Gliadkovskaya, 2021). Furthermore, in pursuit of maximum variation sampling of exercise modalities (Sandelowski, 2000, 2010), potential participants were also required to regularly engage in muscle-strengthening exercise, aerobic exercise, or both. A “regular exerciser” was defined as meeting the national recommendations for muscle-strengthening physical activity (targeting all major muscle groups at least twice per week; (Piercy et al., 2018) or participating in at least moderate aerobic exercise on three or more days per week for the prior three months, following criteria established by the American College of Sports Medicine’s pre-participation screening process (Liguori, 2020).

#### Interview Guide Development and Piloting

Interviews followed a semi-structured format, providing flexibility through a defined set of key questions used to guide discussion. The interview guide was organized into three sections: (1) motivations for using wearable technology (e.g., “Based on all the wearable devices on the market what made you pick the Oura™ ring/ Whoop™ band?”), (2) interpretation and daily use of feedback (e.g., “These devices are designed to give you either a readiness score or recovery score. What does this mean, to you?”), (3) alignment or discrepancies between feedback and subjective experiences (e.g., Think about your recovery/readiness score given by your Oura™ ring/Whoop™ band and your own subjective/personal perceptions of recovery/readiness. Can you talk about a situation when these didn’t line up?”). This approach allowed both the interviewee and interviewer to explore ideas and responses in greater detail, facilitating the discovery of participant-driven insights (Kallio et al., 2016). To enhance positionality and identify any potential biases within the interview guide, mock interviews were conducted with the research team and with three individuals who met the inclusion criteria, ensuring that the questions prompted detailed responses and captured diverse perceptions of the phenomenon of interest (Sampson, 2004). For access to the complete interview guide, readers are referred to Ibrahim et al. 2024(b).

#### Interview Schedule

 Prospective participants expressed their interest in the study by contacting the lead author, who then conducted an informational session via Zoom, where the purpose and criteria were explained. Individuals who met the criteria and provided verbal consent were familiarized with the interview process and were scheduled for an interview within the subsequent two weeks. To ensure anonymity, each participant selected a pseudonym, which was used throughout the recorded interview. Interviews were conducted between December 2022 and March 2023, with durations spanning from 36 to 92 min. No incentive or compensation was offered for participation. Interviews were transcribed verbatim using Zoom’s automated transcription feature and manually cleaned for accuracy. Transcripts were sent to each participant within 5–7 days for member checking, which confirmed transcript accuracy without conflicts.

### Data Analyses

Once data saturation was reached—defined as the point at which sufficient data had been collected for replication, and any new data no longer contributed meaningful insights (Fusch & Ness, 2015)—a reflexive thematic analysis was conducted using interview transcripts (Braun & Clarke, 2019; Braun et al., 2018). This approach prioritizes transparency, awareness of theoretical assumptions, and avoids using predetermined codes or coding reliability, employing an inductive and iterative process of repeated reading, reviewing, and refinement of codes and themes, without being restricted by prior theories or frameworks.

The six Whoop™ band participant transcripts were chosen and were independently analyzed by all authors. Themes were then generated from meaningful points in the data by the first author. An analysis session was used to identify commonalities and differences, during which the second and third authors provided feedback as ‘critical friends’ to encourage exploration and reflection of alternative explanations and interpretations (Rankin, 2017; Smith & McGannon, 2018). The resultant framework was subsequently refined and applied to the remaining eleven Oura™ Ring participants. The entire analysis process was conducted manually without the assistance of a software package. Furthermore, we sought the expertise an ‘expert qualitative researcher’ (Cutcliffe & McKenna, 2004) who served the role as an external auditor (Creswell, 1998). This collaboration led to the division of our finding into two manuscripts to allow for the necessary attention and space for a comprehensive examination of resultant themes.

## Findings

Full details regarding the participant characteristics and the initial three themes can be found in the previous publication (Ibrahim et al., 2024b). A total of 25 participants initially expressed interest in participating; however, two were excluded from the study due to insufficient engagement with their COTSW, and six declined to participate following screening. Recruitment was discontinued once data saturation was achieved, which occurred with 17 participants (35% male, 82% white, mean age 32.3 ± 4.9, 53% holding advanced degrees, 71% with an income of $80,000 or more). For the current study, Theme 1 highlights the personal validation respondents derive from the data, empowering them to engage in self-discovery activities pertaining to training and non-training related responses (Theme 2). Theme 3 suggests the potential for undesirable reactivity to the provided metrics given by the COTSW. The following section presents these themes and related subthemes in detail, utilizing ‘in-vivo’ quotes from our sample (*signifies Oura™ users, ^†^ signifies Whoop™ band users).

### Theme 1. ‘It’s Kinda Validating, Right?’ (Detroit^†^)

#### Subtheme 1.1.‘Reinforces My Healthy Habits’ (Powerade^†^)

 Participants expressed how awareness of the metrics provided from their devices reinforced their habits and provided positive feedback on their lifestyle choices. For many participants, the positive feedback assisted them with *‘removing their ego’* and *‘actually listen’* to the device helping them stay disciplined and motivated with their training. Sean^†^, for example, indicated *‘the discipline of training it’s months and months of work to sometimes get a result… even a little bit of something like a metric to see I do this*, *and this happens can help. It’s that positive feedback.’* The *‘data driven’* insights also provided *‘justification’* that their behaviors were impacting their scores as Brian^†^ states, ‘*it gives me sort of an affirmation and confirmation that what I’m doing around my recovery process is working. So*, *for example*, *when I hit the metrics how I want to my perception of recovery it’s sort of justified and then I feel better*, *because I know that I’m impacting it.’* Additionally, the data-supported validation played a crucial role in helping participants check their behaviors and make necessary adjustments: LC^*^ *‘Yesterday I did more restorative work*, *some yoga*, *stretching*, *went for a walk*, *I did not train*, *and then I did a meditation class last night and I went to bed early. I was like I have to [expletive deleted] do this today because I am literally losing my mind up for not sleeping well*, *and then bam I had a great readiness score today [laughter]. So*, *it’s like that reinforcement*, *or helps me check myself.’*

#### Subtheme 1.2.‘It Helps Validate My Feelings’ (Misty^†^) 

In this subtheme, participants found objective scores and metrics valuable in validating their subjective feelings about their mental and physical states. They recognized and appreciated how *‘powerful’* quantifiable data was in ‘*affirming’* and ‘*confirming’* their perceptions of well-being. Quantifying and tracking their feelings through the data was seen as an empowering tool and provided a tangible way to be *‘in sync’* with their subjective experiences with objective measurements. Sean^†^, for example, noted *‘It can quantify things I’m feeling*, *or it can give me a trend… it can help give some context on how I’m feeling.’* Participants acknowledged that the metrics did not dictate their decisions but rather supported their existing knowledge and instincts about their bodies and acted as a *‘safeguard’* against overexertion and reverting back to *‘past tendencies.’* As DN^*^ explained, *‘I like seeing the numbers*,* and I don’t feel like I let it dictate what I do*, *but at the same time without it*, *because I’m so used to it*, *I think I’d feel lost. I know my intuition. I would know if I’m tired. I would know if I need to take a rest day*, *but something about it telling me it validates how I’m already feeling.’*

### Theme 2. ‘It’s Really Putting the Power in My Hands’ (Misty^†^)

#### Subtheme 2.1.‘What the [Expletive Deleted] was That?’(Powerade^†^)

 In this subtheme, participants revealed experiencing moments of surprise and curiosity when analyzing their scores from their devices. They often questioned sudden *‘spikes’* or *‘drops’* in metrics and decided to *‘dive deeper’* to understand the reasons behind the *‘black box’* score given.

*Powerade*^†^: *The other day it was interesting that I had to tape probably 5 or 6 people in a row*, *and I noticed that looking at my heart rate data across the day*, *and I see that there was like this massive spike*, *and I was like what the hell was that? And I like to figure out what it was based on what my day was like. I was like*, *oh*, *yeah*, *that’s because I had to like tape 6 kids really quickly that makes sense.*

Some elaborated on developing rituals to analyze their data as *‘sometimes is comes down to like that journaling piece and reflecting… and you know now that makes total sense’* (Sean^†^). Similarly, Brian^†^ states, *‘[My] morning ritual routine is checking through my recovery*, *and then adding my journal entries in from the previous day judging did I drink caffeine? What time was that? Did I take vitamin D? And then if that number is lower than I expected*, *then I want to know why and I try and figure it out. Okay*, *let’s look at the metrics. Was my heart rate variability low? Did I have increased respiratory rate? I try and figure out was there anything different about yesterday? Or is it just a case of last night [I] didn’t get enough sleep? So*, *that’s my process*, *learning to understand what that number means*,* what happened yesterday that is impacting it.’*

#### Subtheme 2.2.‘If I have Something New, I Want to Try I May See if it Affects the Data’(Powerade^†^)

 Within this subtheme participants proactively engaged in experimenting new lifestyle adjustments and recovery tools to observe their effect on their scores provided by their wearable devices. Some participants viewed the data as a ‘*game*, motivating them to push their boundaries in hopes in improving their overall fitness; *‘A fun thing*, *when you get numbers*, *you can make a game out of it. It’s how much more can I do [laughter]. So*, *if I have a green score doing a workout of this much is optimal [places hands at chest height]*, *but this one promotes a little bit more fitness [places hands at chin level] if I can recover. Well*, *let’s see if I can do that’* (Sean^†^). In contrast, other participants like Rose^†^ were surprised and reluctant to accept the data’s support during their experiment process stating ‘*this month of January*, *I’m doing no caffeine*, *no sugar*, *no alcohol*, *and my sleep has been the best it’s ever been*, *and I hate [laughter] that the data is right. I hate that doing these healthier [air quotes] protocols actually does improve your sleep.’*

#### Subtheme 2.3.‘I’m More in Tune with my Body Now’ (Misty^†^)

 Through prolonged utilization with their wearable participants experienced a transformative process as their *‘brains changed so much’* allowing for a heightened sense of self-awareness and a deeper connection with their bodies. Brad^†^ describes this effect stating, ‘*this is pretty much to the point where I know how I feel even if I were not to be wearing it.’* Letty^*^ emphasized this process by *‘looking at that score and thinking*, *okay*, *I have an 80 readiness today*, *how do you actually feel at 80 out of 100 and does that actually match how you’re subjectively feeling today?* Interestingly, participants emphasized the evolving nature of this process, acknowledging that these wearables will ‘*eventually run their course*, while still serving as a valuable tool to *‘keep you in check.’*

### Theme 3. Potential for ‘More of an Emotional Response Rather than a Rational One’ (Brian^†^)

Participants exhibited a complex interplay with each of the wearable’s interfaces. Initially, some expressed concerns about *‘I was going to get so married to these numbers that it was gonna affect me.’* Brian^†^ shared his initial experience as: *‘I used to look at my recovery the first thing in the morning and I became a little bit obsessed about it… I started creating anxiety around that score and if the number was lower*, *I would immediately feel negatively*, *whereas if it was higher*, *I would get this uplifting*, *positive effect from it.’* However, overtime, *‘I have delayed looking at the recovery score first thing in the morning. I didn’t want the first thing that I see in the day [to be] something negative*, *because it was tainting my perception of recovery and my positivity.*

While some participants demonstrated resiliency against this emotional response, Detroit^†^ noted differences in their response compared to their partner: *‘If I felt discrepancies to what my data was saying it wouldn’t necessarily affect me*, *but I really did see that with my partner*, *where it would be something that would be frustrating for her*, *and she would feel like well*, *now I’m not set up to have a great day. Mentally it would really affect her.’* Letty^*^ emphasized the importance of objectivity: *‘people talk about a lot of this*, *like a parallel example is with the scale*, *right? Like getting on the scale and this is how much I weigh today. At the end of the day*, *it’s just a number. Staying objective about it rather than allowing like emotions or your actions throughout the day be tied to that number.’* Similarly, LC^*^ highlights that: *‘A lot of times people who are interested in numbers can become really obsessed and to their detriment*, *right? Basically saying*,* if my score is under 83*, *then I can’t do anything today*, *and now everything’s ruined. When in reality it’s like*, *yeah*, *maybe you feel a little off*, *but looking at that actually made you feel a lot worse. It becomes really*, *really detrimental for people in terms of tracking.*’

In some cases, individuals highlighted the interface as a potential source of reactivity. The quick and intuitive color-coded visual provided by the Whoop™ band, for example, was subject to interpretation as Sean explains ‘*They grade those as red*, *yellow*, *and green and so you’d think*, *because I think with most people’s association well*, *green is good*, *yellow is not that great*, *and red is really bad. But according to Whoops metrics*, *yellow is average*, *and green is excellent*, *or like they would call it peak recovery and red is below average recovery. And so*, *they try to frame it positively*, *but sometimes I think with the color association if I woke up with a red recovery I would think*, *oh*, *wow*, *I’m not doing well and must be sick. Something must be wrong. I would probably start feeling kind of negative about myself.* Emotional nuances extended to feelings of guilt: *‘Sometimes you can have a green recovery score and almost feel like a guiltiness of I don’t want to waste this good recovery score*, *because I don’t know if I’m gonna have a good score tomorrow*,* but that can be dangerous.’* Some participants *‘academic background’* influenced their perceptions of their Oura™ ring scores that are given on a scale of 0-100. Michelle^*^ states that *‘I want to make an 80 or higher… anything lower than that I feel like I’ve failed.’* Rose^*^ reflected on the scoring system, remarking, *‘It’s almost like we get our report card in the morning.’* Sarah^*^ began to question her interpretation stating ‘*I want to get an A or maybe a high B at the lowest. So*, *I think that. I don’t know maybe that’s a me issue [laughter]*, *not an Oura issue. It’s probably my brains misalignment with what the number means.’*

## Discussion

The objective of this paper was to present the remaining themes on how regular exercisers perceived information from their COTSW displaying HRV-derived scores and the resulting actions associated with them. Our findings first document the reinforcing abilities of receiving and examining daily readiness/recovery scores. Additionally, through the examination of these scores, our participants demonstrated curiosity about the objective data provided by their COTSW, further building another layer of understanding. However, despite the positive exploratory actions, participants explained the potential for these data could lead to unintended consequences due to complex reactive responses users may have toward these scores and each wearable’s interface. In the following sections, we explore how these findings can be leveraged to further inform the development and refinement of using COTSW to guide person-adaptive exercise program development and promote this behavior.

In accordance with various behavioral theories, the ability of COTSW scores to provide reinforcement can support users’ regular instigation of healthful actions. At a very basic level, operant conditioning principles conceptualize rewards and punishments as primary drivers of behavior in response to various stimuli (Skinner, 1965). Participants previously described how they deliberately took steps to improve readiness and recovery scores through optimizing sleep, hydration, alcohol intake, and methods to improve body integrity (e.g., cryotherapy, sauna) (Ibrahim et al., 2024b). Subtheme 1.1 in the current study suggests that higher readiness scores validated such efforts, serving as a visual (and thusly, more tangible) reward, which then reinforced these supplementary behaviors in support of continued exercise engagement. Provision of affirming feedback (i.e., positive self-perceptions of readiness and recovery aligned with more favorable scores) should also enhance perceptions of self-efficacy, as purported by social cognitive theory (Bandura, 1998). Self-efficacy, when directed toward a valued task – in this case, managing supplementary behaviors necessary to achieve specific performance outcomes – promotes perceptions of competence, which is a fundamental need for developing more self-determined forms of motivation (Deci & Ryan, 1985). Moreover, dual-process theories suggest that all stimuli, including scores provided by COTSWs, are encoded and influence behavior at both automatic and reflective levels (Evans & Stanovich, 2013). When the scores are favorable and validating, they may strengthen associative learning processes, reinforcing motivation for continued engagement in these supplementary behaviors and exercise participation.

Beyond reinforcement and validation, findings from theme two indicate that users were not passive recipients of data but active experimenters, engaging in self-discovery through reflective feedback and behavioral adjustments. This self-discovery process was facilitated by the continuous data collection enabled by COTSW, which aligns with the principle that effective self-regulation begins with self-monitoring (Korotitsch & Nelson-Gray, 1999). These findings support theoretical frameworks of objective self-awareness suggesting that behavior change is most effective when individuals not only receive prescriptive guidance but also develop an understanding of what to do and why it is relevant to their specific context (Duval & Wicklund, 1972; Karkar et al., 2016). Recent research further supports the value of personalization self-guided experimentation in improving self-regulation and adherence (Fedlmeier et al., 2022). Integrating self-monitoring with reflection-in-action (i.e., react to unanticipated circumstances instinctively, without deliberate or conscious thought), may enhance the development of necessary control competencies. These competencies include heightened interoceptive awareness and the ability to interpret psychological and physiological cues, both of which are essential for adaptive training load management and long-term adherence to health behaviors (Carl et al., 2020; Haible et al., 2019; Sudeck & Pfeifer, 2016) Thus, COTSW-derived scores may serve as a bridge between traditional health education and personalized, data-driven decision-making by facilitating an iterative process of self-monitoring, reflection, and behavioral adjustment, thereby contributing to the broader framework of precision medicine (Collins & Varmus, 2015). Indeed, there is a growing interest in self-experimentation to meet such goals in health contexts (Karkar et al., 2016; Lee et al., 2017; Schroeder et al., 2019).

Extending the data-driven insights provided by the COTSW, self-discovery was naturally enhanced through gamification, as users sought motivation through interactive and competitive elements. As highlighted in subtheme 2.2, participants described their interactions with readiness and recovery scores as a ‘game,’ using their data to challenge themselves and improve performance. This perspective aligns with research demonstrating that gamification enhances motivation by introducing elements of challenge, achievement, and progress tracking (Haddad et al., 2017). However, users also highlighted the potential ‘dangers of this game,’ reflecting concerns from a recent review on gamification in apps and technology. Cheng and colleagues (2019) noted that many technology developers attempting to implement gamification have “treated gamification as a black box” (p.11), failing to take appropriate steps to identify key behavior change mechanisms and the processes through which their interventions are intended to work.

Important concerns extend beyond gamification to the broader design of COTSW interfaces, as highlighted in theme three. Specifically, users of the Oura™ ring exhibited an anchoring bias (Furnham & Boo, 2011), where their initial readiness score disproportionately influenced their perceptions and subsequent behaviors, sometimes overriding subjective well-being. This response aligns with research demonstrating that numerical scores often serve as cognitive reference points, shaping expectations and decision-making processes (Tversky & Kahneman, 1974). The mental number line theory (Dehaene, 2002, 2011), suggest that individuals conceptualize numbers along a spatial continuum, where higher values are intuitively associated with better outcomes, influenced by cultural and contextual factors (i.e., grading systems) (Kohn, 1993), as highlighted by our participants’ ‘academic backgrounds.’ The Oura™ ring’s 0-100 scale likely reinforced this left-to-right numerical framework, leading users to perceive higher scores as optimal and lower scores as signs of underperformance. This perception may persist even when physiological variations are negligible, causing users to interpret any deviation below their personal threshold as failure rather than a natural fluctuation in physiological recovery. Similar effects occur in medical, and financial settings, where individuals anchor on round numbers or culturally significant cutoffs, leading to an overemphasis on arbitrary benchmarks (Englitch & Mussweiler, 2001; Epley & Gilovich, 2006; Saposnik et al., 2016). A similar bias emerged among Whoop™ band users who were influenced by color-coded feedback, a well-documented effect in behavioral research (Elliot & Maier, 2012, 2014). Research suggests that red-coded information can induce threat-based responses, while green-coded feedback reinforces perceptions of success and safety (Hill & Barton, 2005; Kliger & Gilad, 2012). This color-based conditioning, combined with numerical anchoring, may further amplify users’ sensitivity to readiness and recovery fluctuations, shaping their psychological and behavioral responses in ways that extend beyond the intended purpose of the device. Future research should embrace co-production through transdisciplinary research and explore how alternative data visualization strategies may help mitigate anchoring biases and heuristic-driven responses in wearable technology users (Choi & Pak, 2006; Kothari & Wathen, 2017).

## Limitations and Conclusion

Despite the insights gained from this study, several limitations should be acknowledged. First, the sample size, although sufficient for thematic saturation, it was consistent of predominantly white, middle to upper-class, and well-educated individuals (Ibrahim et al., 2024b). As this was a qualitative study, the intent was not to generalize findings probabilistically to a broader population. Instead, the goal was to provide a detailed and context rich understanding of participants experiences, allowing the reader to assess the applicability of these findings (Fossey et al., 2002). Second, this study focused exclusively on two specific brands of wearable devices, which may not represent the full spectrum of COTSW available on the market. Different devices may offer different features, affecting user interaction and experience. Thus, findings might be device-specific rather than applicable to all COTSW. Finally, this study did not assess objective behavioral changes linked to wearable-derived scores, leaving open questions about long-term adherence and actual impacts on exercise and recovery practices. Future research should explore a wider range of wearable devices, assess behavioral outcomes over time, and include more diverse user groups to enhance the applicability of findings (Ibrahim et al., 2024b).

In conclusion, this study provides insight into how regular exercisers interact with wearable devices that generate HRV-derived readiness and recovery scores. The findings highlight the reinforcing effects of these scores, their role in fostering self-experimentation, and the potential for emotional reactivity influenced by wearable interface design. While these devices empower users to make informed decisions about their exercise and recovery, they also introduce psychological biases that can shape behavior in unintended ways. As COTSW continues to evolve, future research should explore ways to optimize data presentation to enhance user engagement while minimizing negative cognitive and emotional responses. Additionally, promoting user education on interpreting and applying these scores effectively may support more adaptive exercise behaviors and improved adherence. Ultimately, COTSW present significant potential for facilitating personalized exercise programming, but their effectiveness is contingent upon thoughtful design and informed user interaction.

## Data Availability

The original contributions presented in the study are included in the article/supplementary material, further inquiries can be directed to the corresponding author.
